# The effects of ecological rehabilitation projects on the resilience of an extremely drought-prone desert riparian forest ecosystem in the Tarim River Basin, Xinjiang, China

**DOI:** 10.1038/s41598-021-96742-5

**Published:** 2021-09-16

**Authors:** Aihong Fu, Weihong Li, Yaning Chen, Yi Wang, Haichao Hao, Yupeng Li, Fan Sun, Honghua Zhou, Chenggang Zhu, Xingming Hao

**Affiliations:** 1 State Key Laboratory of Desert and Oasis Ecology, Xinjiang Institute of Ecology and Geography, Chinese Academy of Sciences, Urumqi, 830011 Xinjiang China; 2 School of Water Resources and Hydropower Engineering, North China Electric Power University, 2 Beinong Rd, Changping, Beijing, 102206 China; 3 Akesu National Station of Observation and Research for Oasis Agro-Ecosystem, Akesu, 843017 Xinjiang China

**Keywords:** Ecology, Restoration ecology

## Abstract

The Tarim River Basin in Xinjiang, China, has a typical desert riparian forest ecosystem. Analysis of the resilience of this type of ecosystem under extreme drought conditions and ecological rehabilitation projects could provide a theoretical basis for understanding ecosystem stability and resistance, and provide new ecological rehabilitation measures to improve ecosystem resilience. We employed a quantitative framework to assess net primary productivity (NPP) resilience, emphasizing four aspects of NPP dynamics: NPP, NPP stability, NPP resistance, and maximum NPP potential. We compared ecosystem resilience across four time periods: before the implementation of ecological rehabilitation projects (1990–2000), during construction and partial implementation of ecological rehabilitation projects (2001–2012), during the initial project stage of ecological rehabilitation (2013–2015), and during the late project stage of ecological rehabilitation (2016–2018). There are three main finding of this research. (1) Mean NPP was increased significantly from 2013 and was decreased from 2016, especially in the main stream of the Tarim River and in the basins of eight of its nine tributary rivers. (2) Ecosystem resilience in 2013–2018 was greater than in 1990–2012, with the greatest NPP stability, mean NPP and NPP resistance, especially in part one of the river basin (the Aksu River, the Weigan-Kuche River, the Dina River, the Kaidu-Konqi River, and the main stream of the Tarim River). Ecosystem resilience in 2001–2012 was lowest when compared to 1990–2000 and 2013–2018, with lowest mean NPP, NPP stability, NPP resistance and maximum NPP potential, particularly in part two of the river basin (the Kashigr River, the Yarkand River and the Hotan River basins). Therefore, part one was most affected by ecological restoration projects. When 2013–2018 was divided into two distinct stages, 2013–2015 and 2016–2018, resilience in the latter stage was the lowest, with lowest mean NPP, NPP resistance and maximum NPP potential, especially in the main stream of the Tarim River. This may be due to unreasonable water conveyance in 2014–2015. (3) Ecological resilience has increased significantly in 2013–2015 after the implementation of ecological water transfer projects, river regulation, and natural vegetation enclosure projects. Ecosystem resilience could continue to increase even more in the future with the continued implementation of reasonable ecological water transfer projects.

## Introduction

Climate change and the intensification of human activities have led to serious external disturbances to ecosystems, including drought^1,2^, short-term climate anomalies^3–5^, lack of resources^6,7^, species invasion^7^, and fire^8^. Ecosystem resilience theory may help us to understand how ecosystems deal with these threats^9–11^, as both scientists and the general public have become deeply concerned about the ongoing resilience of ecosystems to external disturbances^12,13^.

Resilience research has, for the most part, focused on wetland, river, mountain, city, farmland, forest, and cold desert shrub land ecosystems^1–3,5–7^, while there has been little exploration of the resilience of desert riparian forest ecosystems. Desert riparian forests, consisting primarily of *Populus euphratica* (desert Poplar), are found along rivers that run through deserts. The world’s three major *Populus euphratica* forests are located in China: in the Tarim River Basin and Yiwu County, both in Xinjiang Province, and in Ejina Banner in Inner Mongolia, with 90% of *Populus euphratica* forest located in the Tarim River Basin. Desert riparian forests are a valuable natural forest resource that^14^ plays an important role in preventing wind and sand erosion, controlling desertification, maintaining regional economic development and ecological security, protecting biodiversity, and ensuring oasis agricultural and animal husbandry production^15,16^. In recent years, driven by regional population growth and economic development, the large-scale exploitation of water and land resources has led to the year-round cessation of the course of the Tarim River, ultimately causing Lake Titema to dry up, the groundwater level to drop significantly, the desert riparian forest ecosystem to degenerate, and the vegetation to decline^17^. To restore this severely damaged ecosystem, the local governments have invested and implemented various ecological rehabilitation engineering measures with remarkable ecological restoration benefits^18^. Among these benefits has been an increase in the height of the water table near the course of the river^19^, and a certain level of vegetation restoration in some areas^20^. However, full restoration and the natural regeneration of vegetation have not yet been achieved. Therefore, new control measures are required, and understanding the resilience of the desert riparian forest ecosystem to external disturbance is a scientific problem that needs to be urgently solved.

Scholars have used a variety of methods to assess the resilience of different ecosystems. These include quantifying the factors affecting forest ecosystem resilience^21^, comprehensive analysis of soil and water conservancy characteristics, the analysis of land use and climate factors in the ecosystem community^22^, monitoring of soil water content and soil temperature^23^, the response of surface vegetation to precipitation, changes in water use efficiency^24^, and calculating ecosystem resilience using linear regression modeling of the Normalized Difference Vegetation Index (NDVI), drought index (SPEI), and air temperature^3^. These methods incorporate many factors, including climate, hydrology, vegetation, and soil. There is some research that uses in-depth analysis of the effects of vegetation factors on ecosystem stability and resistance^2^. For example, Frazier et al.^25^ analyzed total primary productivity (TPP), Ponce Campos et al.^24^ analyzed the sensitivity of the above-ground net primary production (NPP) of terrestrial ecosystems to altered hydroclimatic conditions to reflect ecosystem resilience, Kahiluoto et al.^26^ and Li et al.^2^ used crop yield data to study the resilience of crops to climatic disturbances, and Li et al.^27^ analyzed gymnosperm resilience using tree ring data. The resilience indices of terrestrial ecosystems used in each of these studies was related to the productivity of surface vegetation^24–27^. Based on this earlier scholarship, this paper considers the productivity of desert riparian forests. Tree ring data is only suitable for trees, and not for shrubs and herbs. Given that desert riparian forests are composed of trees, shrubs, and herbs, tree ring data cannot fully reflect the productivity of desert riparian forests. Primary production (PP) is defined as the quantity of products that an autotroph produces through photosynthesis or chemosynthesis^12,28^. Net primary productivity (NPP) is TPP minus consumption by autotrophs for photosynthesis or chemosynthesis^12,28^, and includes consumption by autotrophs^29^. NPP, therefore, reflects the productivity of the plant community under natural environmental conditions^29^. Thus, in this research, vegetation NPP was selected to assess ecosystem stability and resistance to reflect desert riparian forest ecosystem resilience. In other words, NPP is a measure of the productivity and eco-environmental quality of vegetation under natural conditions^28^, climate warming can directly affect the NPP of terrestrial ecosystems through photosynthesis, as well as indirectly through soil absorption^30^. Therefore, the impact of vegetation on ecosystem resilience is generally expressed according to vegetation NPP^30^.

To understand external environmental stress, scholars have focused on climate change stress^1–5^ and species invasion stress^7^, while there has been little focus on the integrated impact of climate change and human activities.

In recent decades, the Tarim River Basin has both suffered from extreme drought and benefited from ecological management^31,32^. Ecological management has changed water demand and supply in the desert riparian forest^32^, affecting vegetation growth^18^. Understanding how ecosystem resilience changes under these two environmental stresses is a key issue in the restoration of the damaged ecosystem. To help understand this change, we propose a framework to compare the impacts of ecological management on NPP resilience by referring to the research of Li et al.^2^, which explored agro-ecosystem resilience by analyzing yield stability and resistance according to yield change. In the case of this current research, agricultural yield was replaced with NPP. There are four parts to the framework: (1) statistical analysis of NPP and NPP trends, (2) NPP stability, (3) NPP resistance, and (4) maximum NPP potential. Statistical analysis of NPP and trends over time can reveal warning signals of change in the state of the system resulting from climate change and management-induced changes to ecosystem processes that might impair or improve long-term resilience. NPP stability measures the ability of the desert riparian forest ecosystem to maintain consistent NPP over time within normal environmental variation, such as temperature and water resources fluctuations. NPP resistance reflects the ability of desert riparian forest ecosystems to mitigate the risk of NPP decline due to extreme drought, while maximum NPP potential measures the ability of systems to produce high NPP under optimum conditions. Based on the research of Li et al.^2^, the larger the mean NPP, NPP stability, NPP resistance and maximum NPP potential, the greater the ecosystem resilience. This approach provides a strong foundation for future analysis of resilience across different ecological management types and provides insights into the design of sustainable ecological management measures that go beyond NPP maximization to consider resilience.

## Study area and method

### Site description

**Figure 1:**
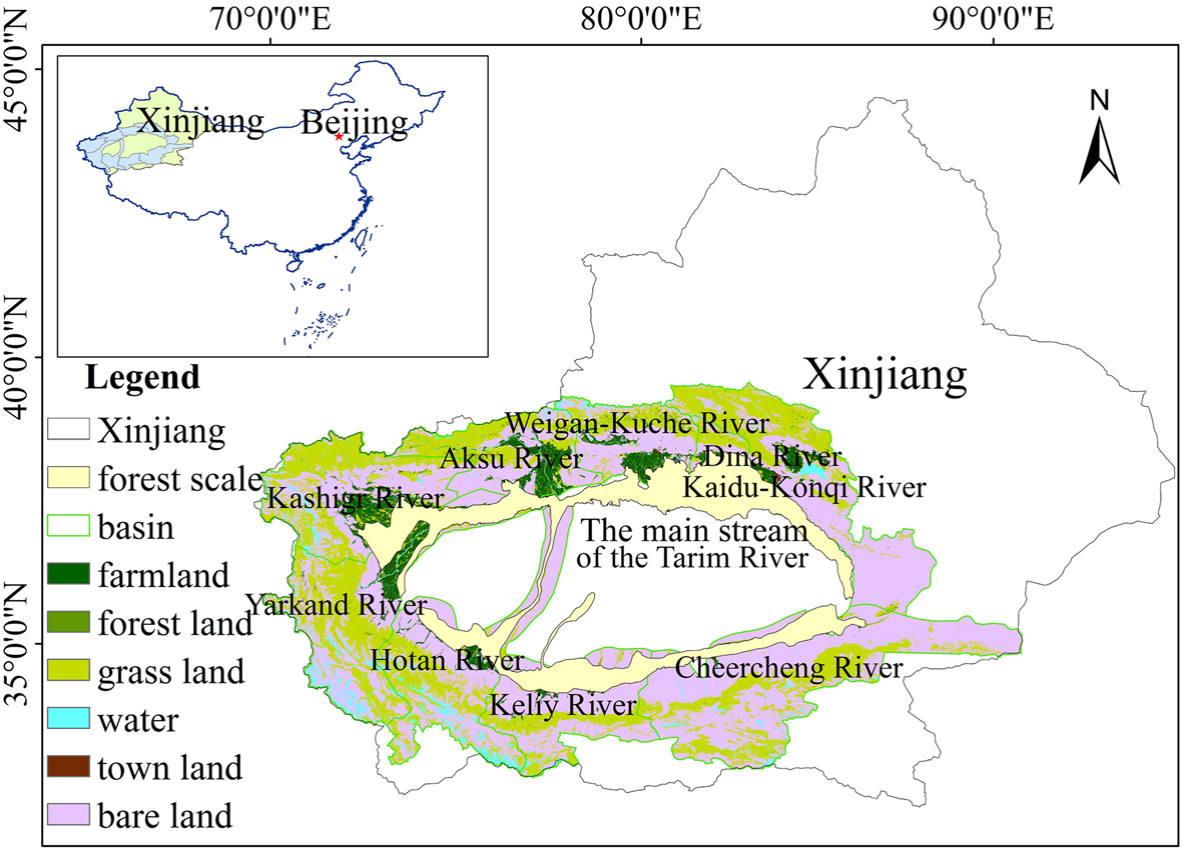
Distribution map of the Tarim River Basin desert riparian forest, Xinjiang, China.

The desert riparian forest of the Tarim River Basin (Fig. 1 (ArcGIS 10.2.2 software, URL: https://www.esri.com)) is located in southern Xinjiang Uygur Autonomous Region, China, and has an area of 11.4 × 10^4^ km^2^. The Tarim River Basin is composed of the basins of the Aksu River, the Yarkand River, the Hotan River, the Kaidu-Konqi River, the Weigan-Kuche River, the Kashigr River, the Dina River, the Keliy River, the Cheercheng River, and the main stream of the Tarim River (Fig. 1). Due to differences in topography and geomorphology, the desert riparian forest is mostly distributed in the middle and lower reaches of each river basin and along the entire main stream of the Tarim River^33^. According to existing literature, there is no evident desert riparian forest boundary between the upper, middle, and lower reaches of these tributaries, so a comparative analysis of ecosystem resilience between the upper, middle, and lower reaches of each river basin was not conducted. However, spatial variability for desert riparian forest as a whole was explored. Land use data were derived from the Global Land Cover Characterization from the International Geosphere-Biosphere Program (IGBP) (http://nsidc.org/data/ease/ancillary.html#igbp_classes) in 2018. From these data, a routine integrated classification of land use/cover change (LUCC) characteristics was obtained based on feature fusion processes. The landscape is composed of 45% barren land, 35% grassland and 16% farmland, urban land, and water. Although only 4% of the land is forested, forest is very important to the maintenance of ecosystem service functions. Among the dominant constructive species are *Populus euphratica, Tamarix ramosissima* and *Alhagi chinensis*. Between 1990 and 2018, the area of desert riparian forest reduced by a significant 1.78 × 10^4^ km^2^ due to a combination of drought and economic development^34^.

The Tarim River Basin has a temperate continental climate characterized by little rain and strong evaporation. In the early Tertiary Period, around 22 million years ago, the collision of India with the Eurasian Plate and the subsequent uplift of the Tibetan Plateau changed atmospheric circulation and geographic patterns in Asia, forming the basic topography of the Tarim River Basin^35^. The current desert environment in southern Xinjiang was formed during the last glacial maximum (20,000–14,000 years ago)^35^. Average annual precipitation is 17.4–42.8 mm, the evaporation capacity is 1800–2900 mm, with a maximum wind speed of 40 m/s, the annual average temperature is 10.7 °C, with a maximum temperature of 39–42 °C, and 2550–3500 of sunshine hours.

The primary water resources in the Tarim River are glacial and snow meltwater from the alpine area to the west. This water flows through the oasis and the desert riparian forest before finally disappearing into the desert or terminal lakes. Water resources, therefore, are incredibly scarce. With an increase in population from 8.26 million in 1998 to over 10.61 million in 2018, and attendant socio-economic development, water from the ecosystem is increasingly used in production and urban living. Climatic conditions have caused the river course to be truncated, and there has been significant decline in the natural vegetation in the lower reaches of the basin. In 2001, the local government implemented conservation and restoration projects for desert riparian forests along the Tarim River Basin to alleviate the serious and on-going degradation of natural vegetation. These projects include ecological water conveyance, river regulation, and natural vegetation enclosure projects^20,32^. River regulation has reduced water loss due to leakage^20,32,36^, while the loss of vegetation due to human and animal impacts has been reduced, and the natural restoration of vegetation has been promoted through natural vegetation enclosure projects^37^. From 2001 to 2018, an ecological emergency water transfer project was implemented in the lower reaches of the main stream of the Tarim River^38^, with a cumulative discharge of 7700 million m^3^. The largest average discharge of 759 million m^3^ occurred in 2011–2012. The quantity of ecological water to promote the restoration of the desert riparian forest has been increased^39^, and the groundwater level has risen^40^ through ecological water conveyance in the lower reaches of the main stream of the Tarim River. The construction of these projects was completed by the end of 2012^18^. Therefore, 2001–2012 was a period during which some projects were constructed and others were implemented. From 2013, all projects were formally implemented, resulting in 7471 km of canal seepage control, 824.60 km of water conveyance dams to harness the main stream, 37 km^2^ of farmland closed to cultivation and 3590 km^2^ of protected forest and grasslands^18^. Previous research has found remarkable ecological restoration benefits following the implementation of part of these ecological rehabilitation projects^18^. However, the concept of NPP has not yet been applied to an analysis of ecosystem restoration efforts.

### Date and methods

The desert riparian forest of the Tarim River Basin has suffered both negative stress (extreme drought) and positive stress (the implementation of ecological rehabilitation projects). We took 1990–2000, 2001–2012, and 2013–2018 as before intervention, during the construction period, and after the implementation of ecological rehabilitation projects, respectively, to explore the change, stability, and resistance of NPP as a reflection of ecosystem resilience.

#### Data collection

The Light Utilization Ratio Model was used to calculate NPP from 1990 to 2018 (see^41^ for the specific calculation method). Climate data, including air temperature, sun light intensity, and sunshine hours, were derived from the Chinese Meteorological Science data-sharing service network (https://data.cma.cn/). Normalized Difference Vegetation Index (NDVI) data for the relevant counties from 1990 to 2018 were retrieved from the Climatic Research Unit (http://www.cru.uea.ac.uk/web/cru/). Groundwater depth data in the middle and lower reaches of the main stream of the Tarim River in 2000–2010 were obtained from long-term field monitoring by our research team. The location map of the monitoring wells is shown in Supplementary Figure S1. Groundwater depth monitoring wells distributed in the lower reaches of the basins of the Aksu River, the Hotan River, the Yarkand River, the Kaidu-Konqi River, the Weigan-Kuche River, the Kashigr River, and the Dina River were set up in 2018. At the time of writing, there are only three years of continuous monitoring data, so these cannot be used to explain the effect of groundwater depth on ecosystem resilience. Groundwater depth change in the middle and lower reaches of the main stream of the Tarim River (see the box area in Supplementary Figure S1) was analyzed to reflect the impact of groundwater depth on ecosystem resilience.

#### Mean NPP and trends

All statistical analyses were performed using SPSS Statistics 12.0, SigmaPlot 12.0 and Microsoft Excel 10.0. Long term mean NPP for the three periods were analyzed using a linear mixed-effects model with NPP as the fixed effect and year as the random effect. Post-hoc Tukey multiple comparisons of means were applied to compare mean NPP across the three periods with confidence intervals adjusted using the Sidak method^2^. The three periods were allowed to have different within-group variances in the model to account for the non-homogeneity of variance. The assumption of normal distribution of residues was verified with the Shapiro–Wilk normality test^2^.

#### NPP stability

Four NPP stability metrics per period were calculated and compared: (1) NPP range, (2) coefficient of variation (CV), (3) NPP variance, and (4) Finlay-Wilkinson (FW) regression slope^2^. NPP range represents the range between the highest and lowest NPP of each period. The other three stability metrics were obtained based on de-trended NPP data (i.e., residuals from regressing NPP against year with period-specific intercepts and slopes) to remove potential biases from NPP increases associated with the implementation of ecological rehabilitation projects^2^. The CV was calculated by dividing the temporal standard deviation by the mean NPP. NPP variance represents temporal variance over the three periods. FW regression slopes were obtained by regressing the de-trended NPP of each period to the environmental index (EI)^2^. EI is expressed as the average of annual de-trended NPP over the three periods and is used as an indication for the overall NPP ability at the respective environmental condition^2^. Periods with smaller NPP range, CV, NPP variance, and FW slopes indicate higher NPP stability^2^. The overall NPP stability of each period was ranked based on the mean stability rank for the four stability metrics^2^.

#### NPP resistance

NPP resistance is a key property of resilience and represents the ability of systems to avoid NPP failure under stressful conditions^2^. NPP resistance was calculated using two metrics: (1) probability of NPP failure based on frequency distributions, and (2) predictions of minimum NPP according to EI^2^.

Probabilities of low NPP were performed by estimating the probability densities of NPP in each period. The probabilities of the three periods to achieve low NPP (< 10th percentile of the pooled NPP distribution estimate) were extracted^2^. The significance of the probabilities of low NPP was determined by comparing each period to the probabilities of low NPP from 1553 randomized NPP sets. The pseudo-p for low NPP probability represents the percentage of times that each period would have NPP lower than the distribution of randomized NPP using a left-tail test^2^. The second method compared the predictions of minimum NPP in the three periods under unfavorable growing conditions (lowest EI) based on a linear mixed-effects model with EI and system as fixed effects and block as the random effect^2^. To indicate actual NPP ranges, de-trended NPP was re-centered to the mean NPP of each period (i.e., adding mean NPP to de-trended data).

#### Maximum NPP potential

Using the same method as NPP resistance, we estimated the probabilities to obtain high NPP (> 90th percentile of the pooled NPP distribution estimate) in the three periods, and the maximum NPP potential under favorable conditions (highest EI)^2^. This measurement helped to indicate management-induced differences in the potential of NPP to capitalize on favorable growing conditions.

## Results

### Mean NPP and trends

**Figure 2:**
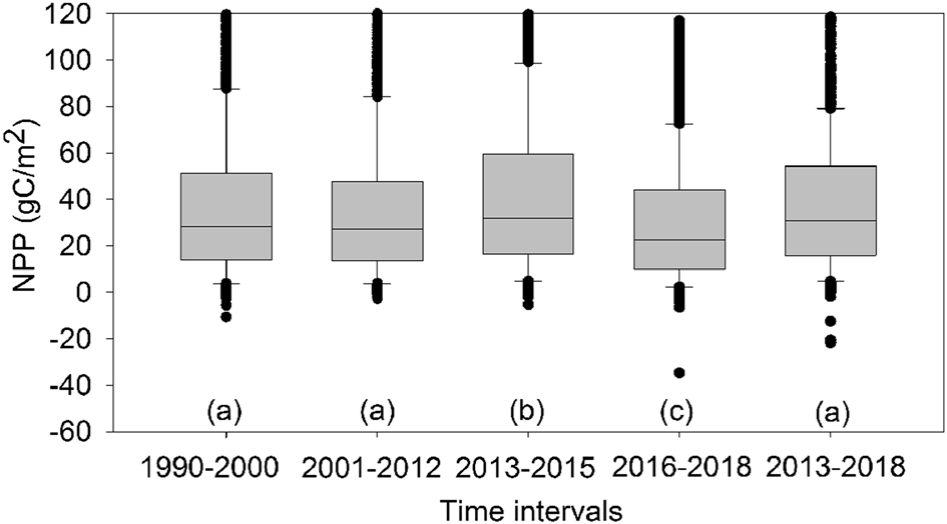
Mean NPP at three time intervals. Letters represent significant differences in mean NPP at the 0.05 significance level.

**Figure 3:**
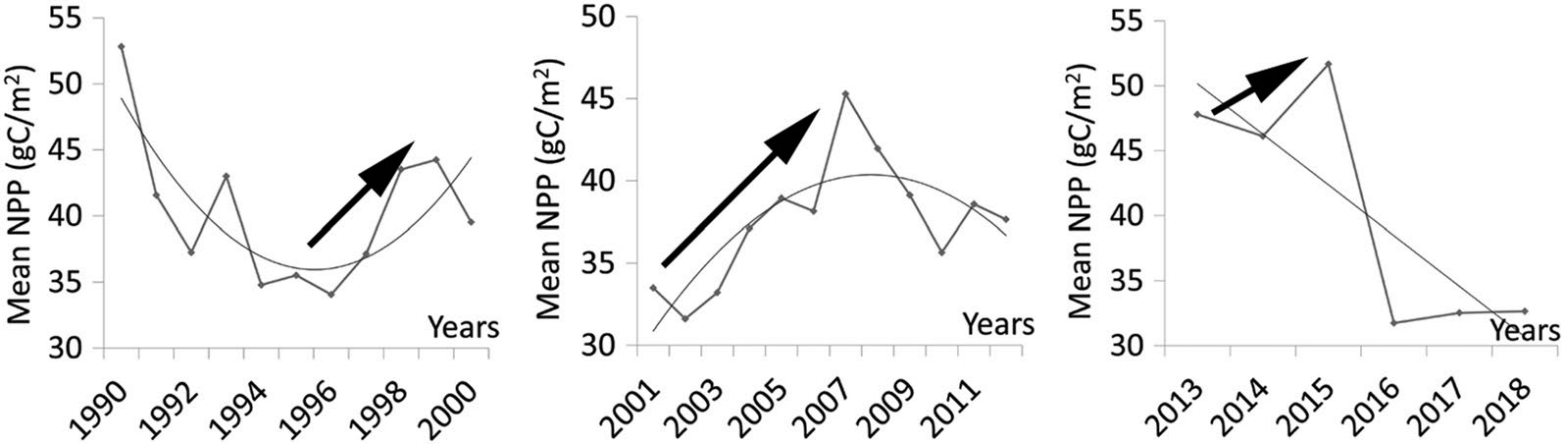
Trend of NPP in (**a**) 1990–2000, (**b**) 2001–2012, and (**c**) 2013–2018.

There was no significant difference in mean NPP between 1990–2000, 2001–2012, and 2013–2018 (F_2, 4659_ = 0.13, Sig. = 0.27), but there was a significant difference between 2013–2015 and 2016–2018 (F_1,3103_ = 12.24, Sig. = 0). There were also significant differences for 1990–2000, 2001–2012, 2013–2018 compared to 2013–2015 and 2016–2018 (F_1,3103_ = 3.51, Sig. = 0) (Fig. 2). This indicates that NPP varied from 2013 onward. NPP was largest in 2013–2015 and smallest in 2016–2018, exhibiting an increasing trend in 1996–1999, 2002–2007, and 2013–2015, and a decreasing trend in the other time intervals (Fig. 3). NPP was decreased in 2007–2013 due to a dry year in 2007–2009, when there was almost no water supply to the ecosystem from the Tarim River. Runoff increased from 2010, but NPP did not recover until 2013^42^. After 2013, NPP was increased rapidly, but fluctuated between 2016 and 2018. Because NPP was increased significantly in 2013–2015, and then was decreased in 2016–2018, we used two separate phases (2013–2015 and 2016–2018) to analyze ecosystem resilience.

**Figure 4:**
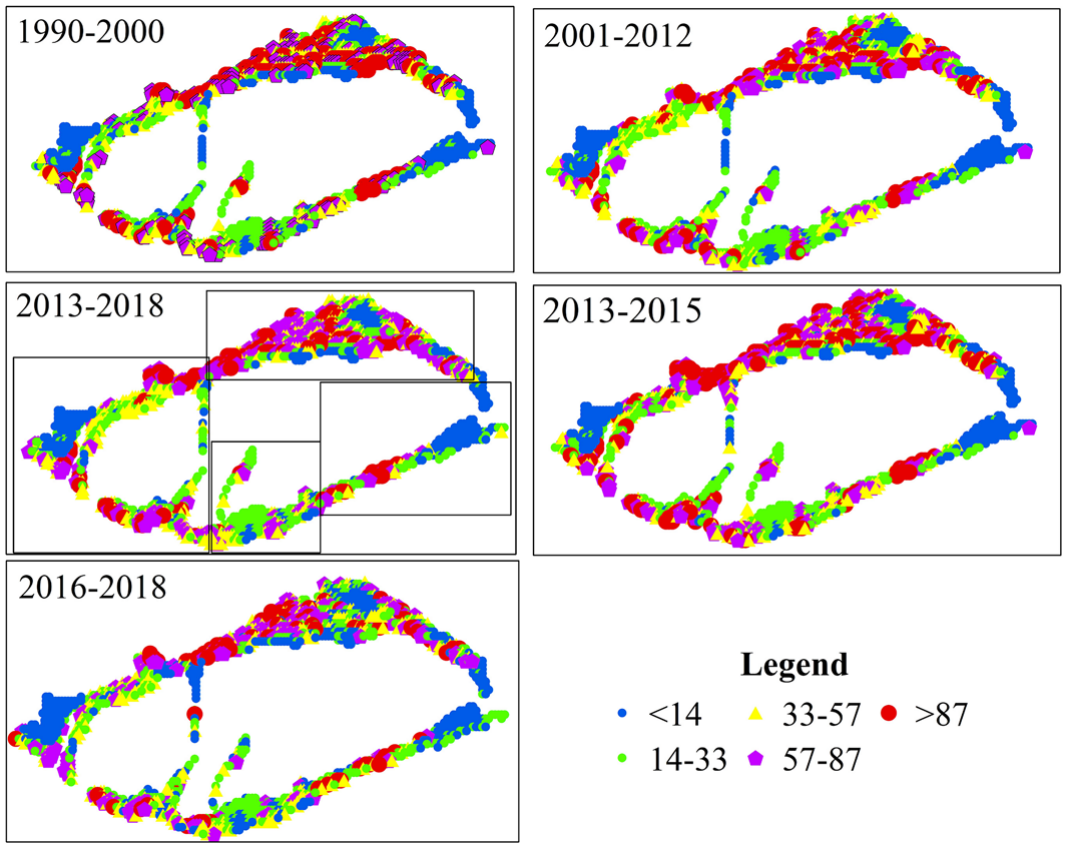
The spatial variation of NPP in the different time intervals.

Spatial variations of NPP in 1990–2018 were analyzed (Fig. 4). According to the geographical location and distribution of each river basin in the Tarim River Basin system (Fig. 1), we artificially divided the desert riparian forest into five parts. Part one consisted of the basins of the Aksu River, the Weigan-Kuche River, the Dina River, the Kaidu-Konqi River, and the main stream of the Tarim River. Part two consisted of the basins of the Kashigr River and the Yarkand River. Part three consisted of the Hotan River basin. Part four consisted of the Keliy River basin. Part five consisted of the Cheercheng River basin. NPP was significantly different between most of the river basins (F_1169_ = 2.61, Sig. = 0). No significant difference (F_1169_ = 2.61, Sig. = 0.14) was found between parts two and three, so these were regrouped into part two. Finally, the desert riparian forests of the Tarim River Basin were divided into four parts.

Further analysis was conducted on variations in NPP in the desert riparian forest in each part during the different time intervals (Fig. 4).

In part one, NPP exceeded 57 gC/m^2^ and was increased significantly in 2013–2018 compared to 1990–2012. NPP was increased at a rate of 29.27% (F_677_ = 2.94, Sig. = 0) in 2013–2015 compared to 1990–2012, and was decreased after 2015 at a rate of 38.69% (F_677_ = 2.94, Sig. = 0) compared to 1990–2012.

In part two, NPP in 2013–2015 was increased by 12.88% (F_539_ = 0.71, Sig. = 0) compared to 1990–2012, and was decreased after 2015 with the bigger decrease rate of 25.32% (F_539_ = 0.71, Sig. = 0) compared to 1990–2012.

In part three, NPP in 2013–2018 was increased significantly (F_238_ = 0.69, Sig. = 0.02) compared to 2001–2012. In 2013–2015, NPP was increased significantly at a rate of 24.08% (F_238_ = 0.69, Sig. = 0.01) compared to 1990–2012, but was decreased in 2016–2018 at a rate of 14.29% (F_238_ = 0.69, Sig. = 0.05) compared to 1990–2000.

In part four, there were no significant differences between the time intervals (F_215_ = 0.12, Sig. = 0.31) with an annual average NPP of 26 gC/m^2^ and, therefore, no obvious increase or decrease in vegetation productivity level over the 29-year period.

### NPP stability

**Figure 5:**
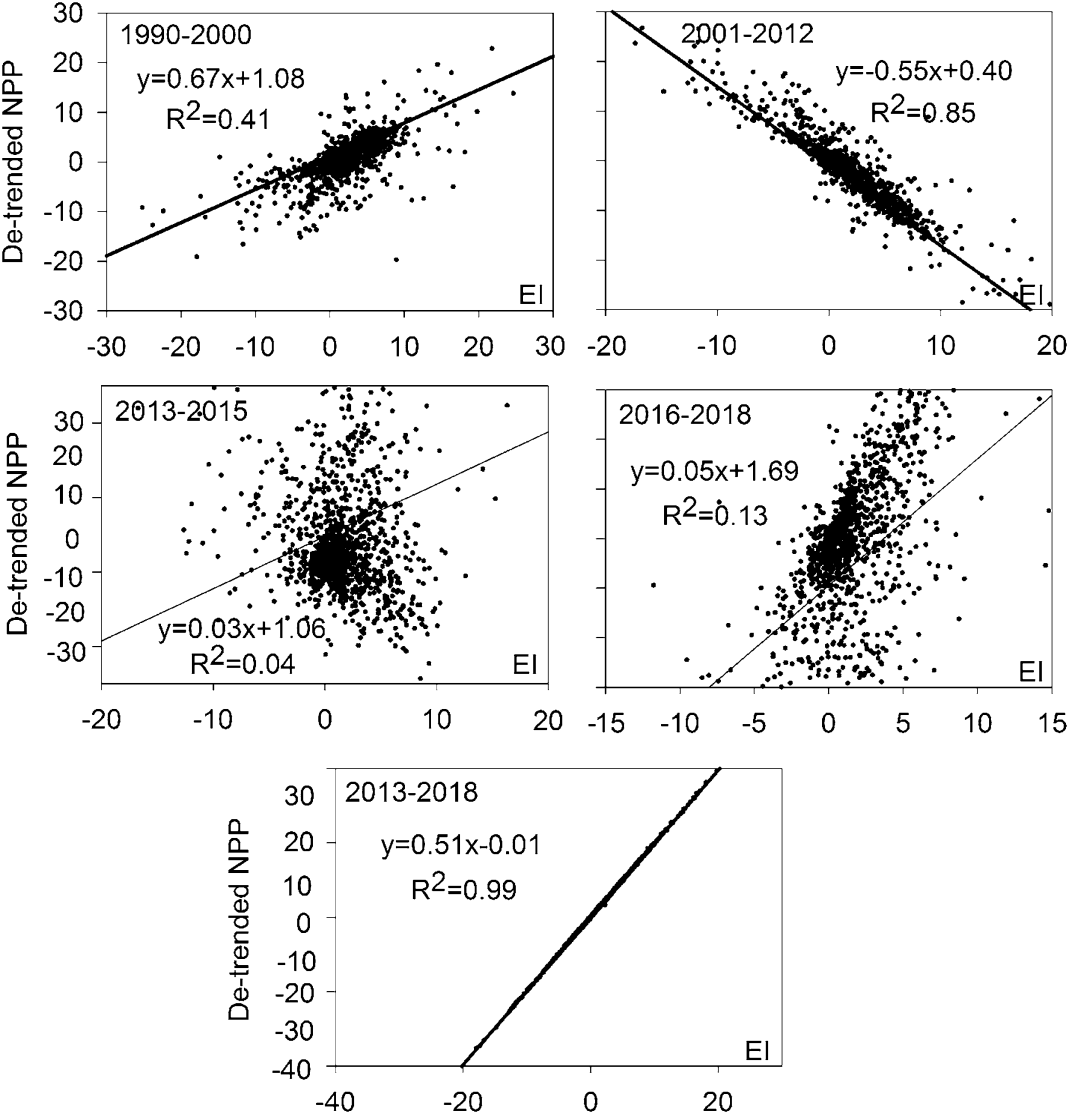
NPP stability by regressing de–trended NPP in the different time intervals against the environmental index (EI) calculated as the yearly mean de–trended NPP.

When 2013–2018 is considered as a whole, NPP during this period was the most stable, with the lowest NPP range, CV and FW slope of the three intervals compared to 1990–2000 according to ranking (Table 1). The FW slope in 2001–2012 was negative (Fig. 5, R^2^ = 0.89), indicating that NPP in 2001–2012 decreased as EI increased, and vegetation continued to gradually degrade alongside improvements to environmental conditions. When 2013–2018 is considered as two distinct stages, 2013–2015 and 2016–2018, the FW slopes in 2013–2015 and 2016–2018 were positive, but non-significant (Fig. 5, R^2^ = 0.04 and 0.13). According to the ranks in the different time intervals, ecosystem stability was the same in 2013–2015 and 2016–2018 (Table 1). The least stable stage was 1990–2000 (Table 1).

**Table 1:** NPP stability parameters and ranks for 1990–2000, 2001–2012, and 2013–2018, and for 1990–2000, 2001–2012, 2013–2015, and 2016–2018.

	Time intervals	NPP stability parameters				
		NPP range	CV	NPP variance	FW slope	Rank
The whole basin	1990–2000	682.41 (2)	6.88 (2)	129.59 (1)	0.82 (2)	1.75
	2001–2012	510.41 (2)	− 6.30 (3)	456.34 (2)	− 0.56 (3)	2.50
	2013–2018	313.13 (1)	6.65 (1)	623.25 (3)	0.51 (1)	1.50
	1990–2000	682.41 (4)	6.88 (4)	129.59 (1)	0.82 (4)	3.25
	2001–2012	510.41 (3)	− 6.30 (1)	456.34 (2)	− 0.56 (1)	1.75
	2013–2015	491.87 (2)	2.47 (3)	6235.66 (3)	− 0.01 (2)	2.50
	2016–2018	267.69 (1)	− 3.92 (2)	9208.68 (4)	0.07 (3)	2.50
Part one	1990–2000	671.74 (5)	0.96 (3)	26,111.07 (4)	0.68 (5)	4.25
	2001–2012	508.16 (4)	0.97 (4)	22,457.41 (3)	− 0.51 (1)	3.00
	2013–2018	289.86 (2)	0.81 (1)	17,208.12 (2)	0.51 (4)	2.25
	2013–2015	486.60 (3)	1.06 (5)	48,328.20 (5)	− 0.01 (2)	3.75
	2016–2018	267.69 (1)	0.89 (2)	10,573.78 (1)	0.06 (3)	1.75
Part two	1990–2000	671.52 (5)	1.22 (5)	22,678.11 (4)	0.99 (5)	4.75
	2001–2012	507.55 (4)	1.10 (3)	19,305.22 (3)	− 0.61 (1)	2.75
	2013–2018	291.05 (2)	0.85 (2)	10,485.78 (2)	0.50 (4)	2.50
	2013–2015	486.55 (3)	1.13 (4)	25,339.42 (5)	− 0.11 (2)	3.50
	2016–2018	117.00 (1)	0.80 (1)	6352.81 (1)	0.14 (3)	1.50
Part three	1990–2000	178.74 (4)	0.74 (3)	5669.25 (4)	1.31 (5)	4.00
	2001–2012	143.65 (3)	0.74 (3)	4185.38 (2)	− 0.61 (1)	2.25
	2013–2018	135.88 (2)	0.61 (1)	3973.49 (1)	0.51 (4)	2.00
	2013–2015	250.59 (5)	0.69 (2)	6469.39 (5)	0.03 (2)	3.50
	2016–2018	132.37 (1)	0.76 (4)	4535.04 (3)	0.11 (3)	2.75
Part four	1990–2000	490.95 (5)	1.74 (4)	25,111.09 (5)	1.18 (5)	4.75
	2001–2012	389.88 (3)	1.69 (3)	17,135.50 (3)	− 0.63 (1)	2.50
	2013–2018	26,264 (2)	1.36 (2)	11,328.61 (2)	0.50 (4)	2.50
	2013–2015	471.00 (4)	1.76 (5)	24,436.54 (4)	− 0.09 (2)	3.75
	2016–2018	125.74 (1)	1.24 (1)	6925.53 (1)	0.09 (3)	1.50

Numbers in parentheses represent ranks of individual NPP stability metrics for 1990–2000, 2001–2012, and 2013–2018, and for 1990–2000, 2001–2012, 2013–2015, and 2016–2018.

### NPP resistance

When 2013–2018 is considered in two stages, 2013–2015 and 2016–2018, ecological management significantly decreased the risk of low NPP, with the lowest probability of NPP failure (< 10th percentile) at 3.30% and 3.00% in 2013–2015 and 2016–2018, respectively, compared to 5.35% in 1990–2000 (Table 2), especially in parts one and three (Table 2). The possibility of high NPP increased non-significantly (Table 2). When 2013–2018 is considered as a whole, ecological rehabilitation significantly decreased the risk of low NPP (Table 2), indicating that the average response of vegetation was favorable in 2013–2018 (i.e., highest EI), compared to 1990–2000.

**Table 2:** The probabilities of obtaining low and high NPP, and the minimum and maximum NPP potential for 1990–2000, 2001–2012, and 2013–2018, and for 1990–2000, 2001–2012, 2013–2015, and 2016–2018.

	Time intervals	Probability of low NPP (< 10th percentile)	Probability of high NPP (> 90th percentile)	Minimum NPP potential (gC/m^2^)	Maximum NPP potential (gC/m^2^)
The whole basin	1990–2000	5.35	1	0.16	671.89
	2001–2012	4.00	1	0.10	507.91
	2013–2018	2.38	1	0.30	291.47
	2013–2015	3.30	1	0.09	486.68
	2016–2018	3.00	1	0.06	164.27
Part one	1990–2000	1.75	1	0.16	671.89
	2001–2012	1.75	1	0.10	507.91
	2013–2018	0.88	2	1.61	291.47
	2013–2015	1.40	2	0.18	486.68
	2016–2018	1.50	1	0.06	164.27
Part two	1990–2000	0.79	2	0.38	215.00
	2001–2012	0.73	2	0.36	217.67
	2013–2018	0.70	2	0.43	178.40
	2013–2015	0.85	2	0.44	300.30
	2016–2018	0.90	2	0.35	115.91
Part three	1990–2000	0.10	2	1.88	180.62
	2001–2012	0.07	2	1.25	144.90
	2013–2018	0.04	2	1.63	137.51
	2013–2015	0.08	2	5.81	256.40
	2016–2018	0.03	2	0.36	126.01
Part four	1990–2000	1.38	2	0.26	480.43
	2001–2012	1.38	2	0.25	387.38
	2013–2018	1.08	2	0.30	260.80
	2013–2015	1.40	1	0.44	465.82
	2016–2018	0.78	3	0.30	125.12

Over the entire basin, vegetation was more resistant to unfavorable conditions in 2013–2018, as indicated by a higher minimum NPP potential (0.30 gC/m^2^), than in 2001–2012 (0.10 gC/m^2^) and 1990–2000 (0.16 gC/m^2^), under the lowest EI (Table 2), especially in parts one, two and four. Minimum and maximum NPP potentials increased briefly and significantly in 2013–2015 compared to 1990–2000, but decreased precipitously in 2016–2018. This suggests that the implementation of ecosystem rehabilitation projects decreased the probability of low NPP, but with the possibility of ongoing fluctuations.

### Ecosystem resilience

**Figure 6:**
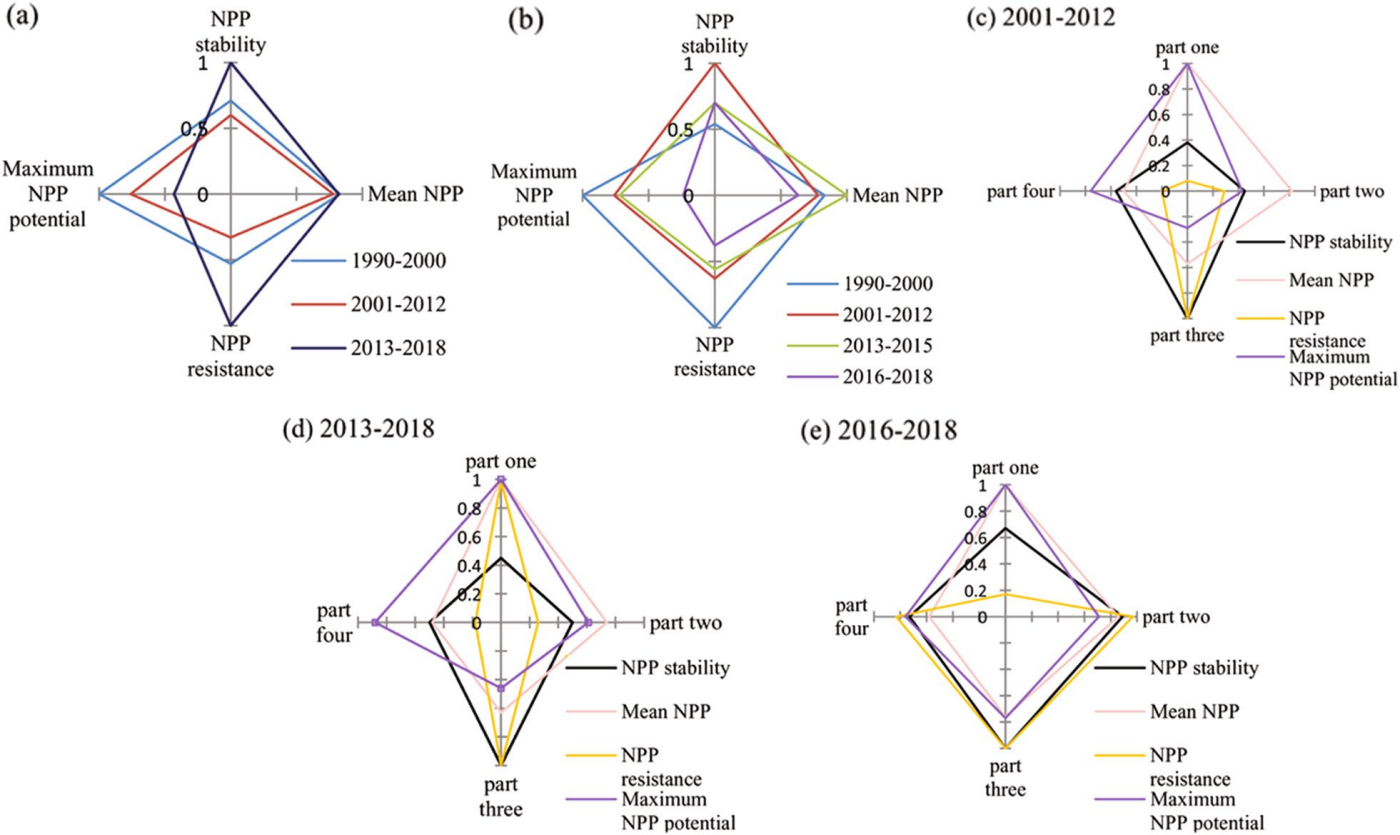
Summaries of NPP resilience in 1990–2000, 2001–2012, 2013–2015, 2016–2018, and 2013–2018, in the different regions. Values represent the ratio of the NPP performance at each time interval to the maximum. NPP stability is the average ratio of four stability metrics (see Table 1). NPP resistance is the ratio of minimum NPP potential (see Table 2).

Ecosystem resilience in 2013–2018 was highest when compared to 1990–2012 (Fig. 6a), with the highest NPP stability, mean NPP, and NPP resistance, especially in part one (Fig. 6d), where the average ratio of the four indicators was highest in 2013–2018. When 2013–2018 is considered in two stages, 2013–2015 and 2016–2018, ecosystem resilience in 2016–2018 was lowest when compared to other time intervals (Fig. 6b), especially in part one (Fig. 6e). Ecosystem resilience in 2001–2012 was lowest when compared to 1990–2000 and 2013–2018 (Fig. 6a), with the lowest mean NPP, NPP stability, NPP resistance and maximum NPP potential, especially in part two (Fig. 6c). Therefore, areas in part one were most affected by ecological restoration projects.

## Discussion

### Resilience assessment method of desert riparian forest ecosystems

The resilience of desert riparian forest ecosystems under the stresses of extreme drought and ecological engineering has been ignored by scholars. Thus far, no suitable method to assess the resilience of desert riparian forest ecosystems had been proposed. Building on the work of Li et al.^2^, this innovative research applied a comparative analysis of NPP resilience to the desert riparian forest before and after the construction of ecological engineering projects. We proposed and implemented an NPP resilience assessment framework, which quantified four core aspects of long-term NPP dynamics: absolute NPP, NPP stability, NPP resistance, and maximum NPP potential. The framework allowed a better integration of resilience and stability metrics into desert riparian forest ecosystem performance assessments based on long-term NPP dynamics and provided a strong foundation for the long-term comparison of ecological engineering approaches. In this paper, the framework successfully identified NPP-specific resilience responses to the implementation of ecological rehabilitation projects.

### The impact of climate change on resilience

An analysis of the correlation between NPP and air temperature, precipitation, sunshine duration, and light intensity was conducted (Table 3). The results show that only the correlation between NPP and sunshine duration was significant in the Tarim River Basin, and non-significant with other climatic factors. However, there was no obvious increase or decrease in annual sunshine duration (R^2^ = 0.04) and NPP (R^2^ = 0.03) in 1990–2015 (Supplementary Figure S2). Therefore, we do not believe that a change in sunshine hours plays a dominant role in NPP and ecosystem resilience.

**Table 3:** Correlation coefficients between NPP and air temperature, precipitation, sunshine duration, and light intensity.

	Air temperature	Precipitation	Sunshine duration	Light intensity
NPP	0.32	0.10	0.72**	0.19

**A highly significant correlation.

### The impact on resilience of water quantity change used for natural vegetation growth

The vegetation in the Tarim River Basin desert riparian forest is composed of trees dominated by *Populus euphratica*, shrubs dominated by *Tamarix ramosissima* and herbaceous plant. The occurrence of young *Populus euphratica* forests is limited to the flood plain and near the river course^43^, and the number is very small, Most are mature and over-mature *Populus euphratica*^44^. Therefore, in this paper, the response of the resilience of young *Populus euphratica* on ecological rehabilitation projects can be neglected.

The vegetation in the Tarim River Basin desert riparian forest has declined considerably under the stress of long-term extreme drought^31,32^. To restore this seriously damaged ecosystem, the local government has implemented a series of ecological management projects^18^. Ecological engineering has varied water management and allocation in the basin, which has made the groundwater depth shallower in the lower reaches of the main stream of the Tarim River (Supplementary Figure S4)^41^. The growth of vegetation is mainly influenced by the groundwater^44^. As the groundwater depth became shallower, average NPP and ecological resilience increased significantly in 2013–2018 (Figs. 2, 6), which showed that the groundwater depth significantly affected the ecological resilience. The ecological restoration benefits of these projects are remarkable^45^. Runoff distribution at the point where the Aksu River, the Kashigr River, the Yarkand River, and the Hotan River flow into the Tarim River, and ecological water conveyance in the lower reaches of the Tarim River exhibited increasing trends in 1990–2018 (Supplementary Figure S3(a, b)), with the exception of 2014. Water consumption for production and urban living decreased slightly but non-significantly (R^2^ = 0.07) in the main stream of the Tarim River in 1990–2016 (Supplementary Figure S3(c)), and the amount of water used for ecological protection increased in the same period (Supplementary Figure S3(d)), with the exception of 2014. Moreover, as of 2015, no ecological water diversion projects have been implemented in the tributaries of the Tarim River, but such projects have been implemented in the lower reaches of the main stream of the Tarim River^46^. The effects of existing projects reached their peak in 2013–2015, after which the effects weakened, and vegetation began to rapidly degrade. This may be one reason for the decline in NPP in 2016–2018. Moreover, there is a lag in the effect of ecological water delivery on the growth of surface vegetation, and ecological water delivery in 2014–2015 only started in summer and missed the vegetation growing season, which may be another reason for the decline in NPP in 2016–2018. In general, increased environmental flow has fostered the rehabilitation of degraded riparian forests. Since 2016, the Tarim River Basin Authority has also carried out ecological water transfer projects in the middle and lower reaches of the tributaries of the Tarim River. Cumulative water discharge from these projects was 52.7 million m^3^ in 2016, 120.8 million m^3^ in 2017, 152.4 million m^3^ in 2018, and 80 million m^3^ in 2019^47,48^. We assume that, if ecological water delivery can be continually implemented in conjunction with the vegetation growing season, the restoration of vegetation will continue.

## Conclusions

The focus of desert riparian forest ecosystem resilience studies must move away from the effects of a variety of comprehensive restoration projects to the prominent influence of single projects within the context of comprehensive projects, such as ecological water conveyance. Our proposed framework of integrating NPP, NPP temporal stability, NPP resistance to unfavorable conditions, and NPP potential to maximize NPP to optimal conditions provides insights for comparing the NPP resilience of different forms of ecological water delivery. Mean NPP, NPP resistance, NPP stability, and maximum NPP potential were greatest in 2013–2018, and especially in 2013–2015. The decline in NPP in 2016–2018 may be due to a lag in the effect of ecological water delivery on the growth of surface vegetation, as ecological water delivery in 2014–2015 started in summer and missed the vegetation growing season. Therefore, the continuing implementation of reasonable water transfer is likely to further promote ecological recovery.

## Supplementary Information

Supplementary Figure S1.

Supplementary Figure S2.

Supplementary Figure S3.

Supplementary Figure S4.
