# Supplementary figures and images for: The effects of ecological rehabilitation projects on the resilience of an extremely drought-prone desert riparian forest ecosystem in the Tarim River Basin, Xinjiang, China
Source: Sci Rep. 2021 Sep 16;11:18485. doi: 10.1038/s41598-021-96742-5 (PMC8445953; doi:10.1038/s41598-021-96742-5)

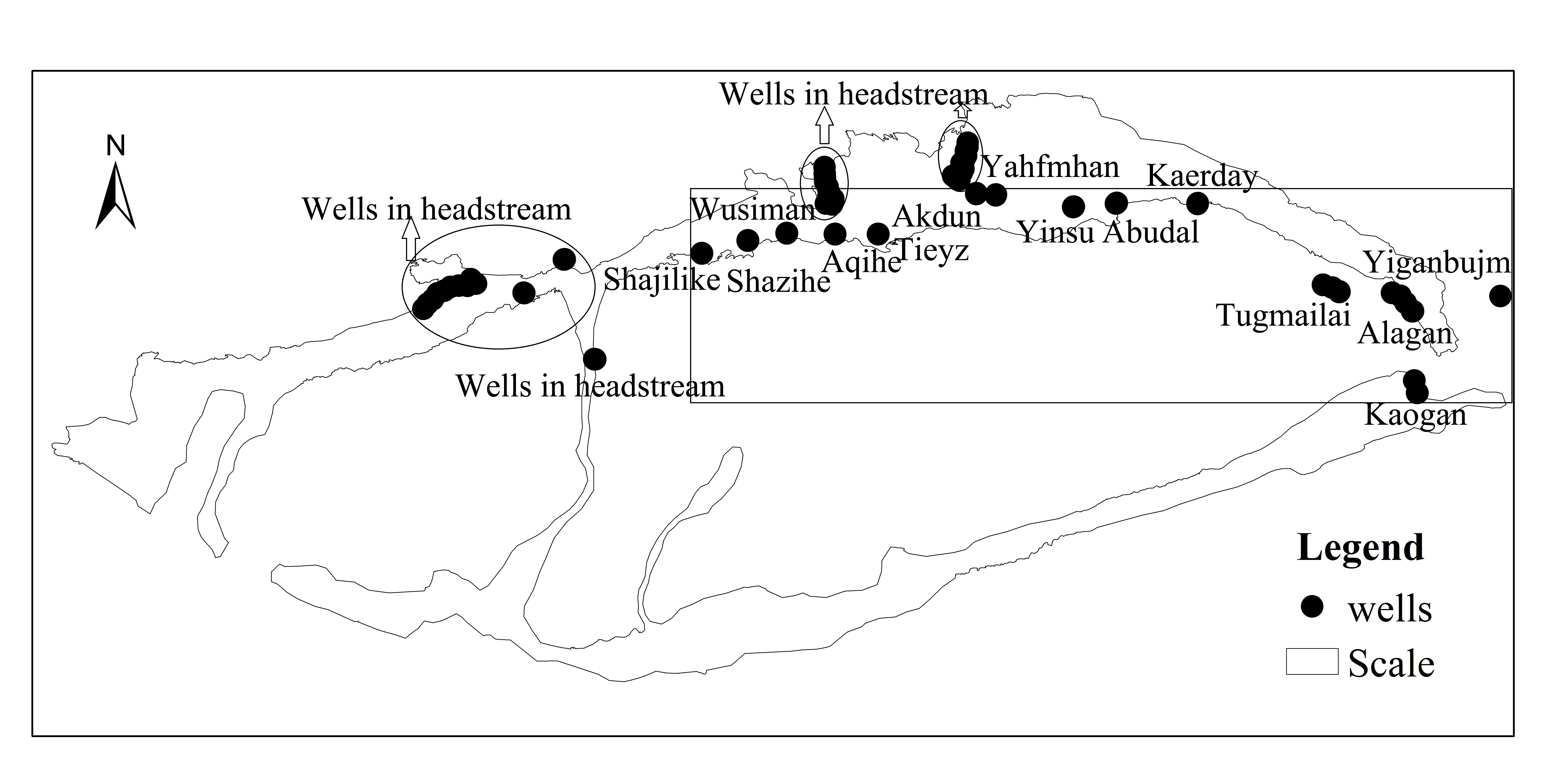

Supplement: Supplementary file 1 — Supplementary Figure S1. [file 41598_2021_96742_MOESM1_ESM.jpg]

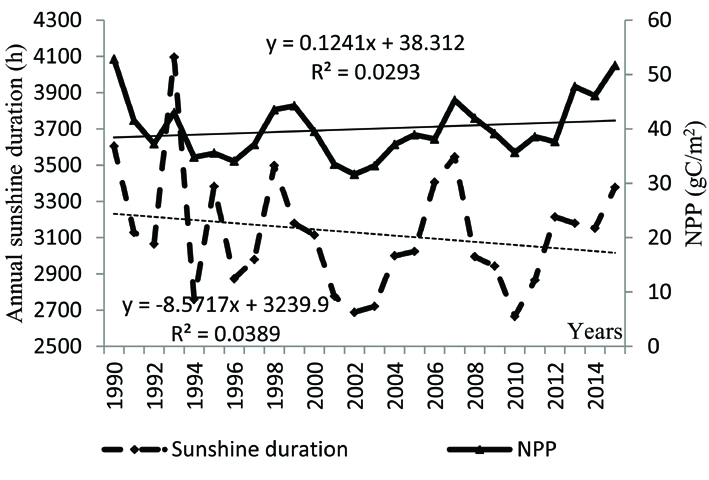

Supplement: Supplementary file 2 — Supplementary Figure S2. [file 41598_2021_96742_MOESM2_ESM.tif]

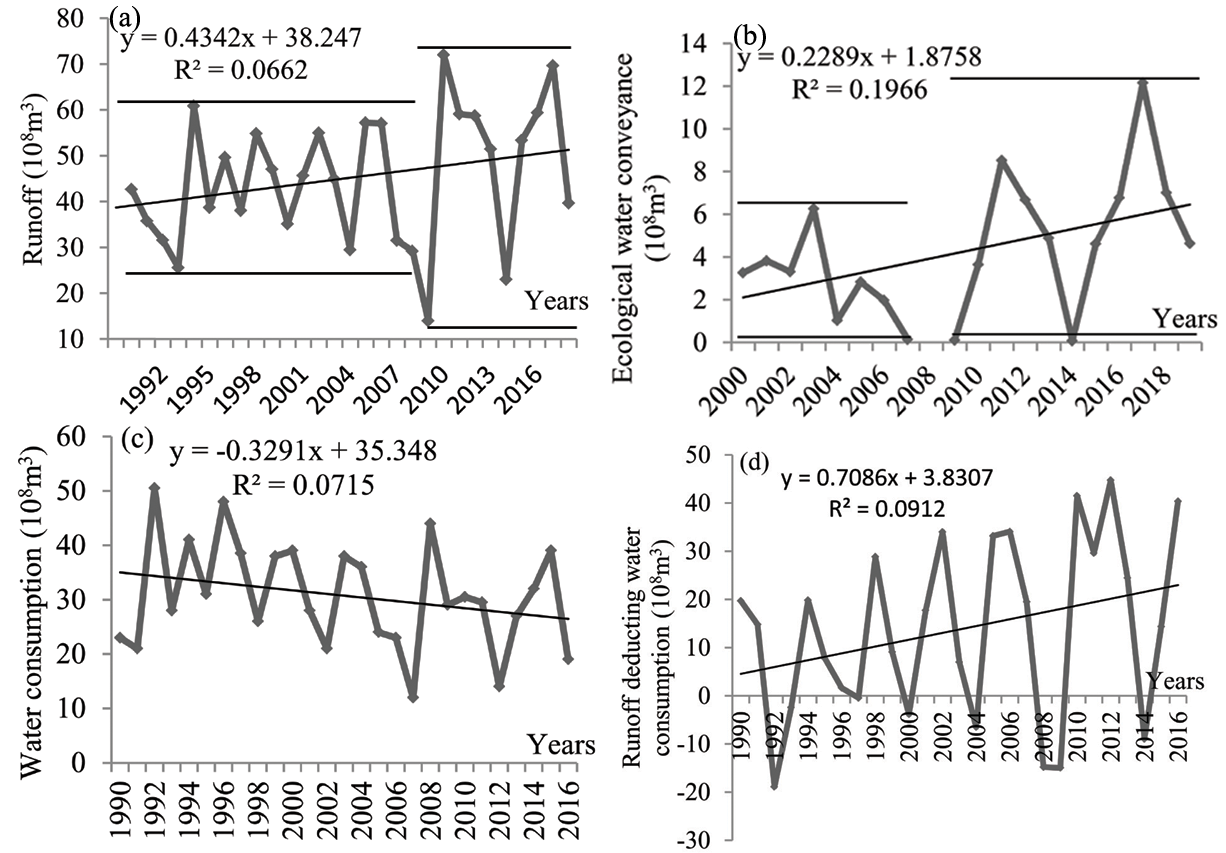

Supplement: Supplementary file 3 — Supplementary Figure S3. [file 41598_2021_96742_MOESM3_ESM.tif]

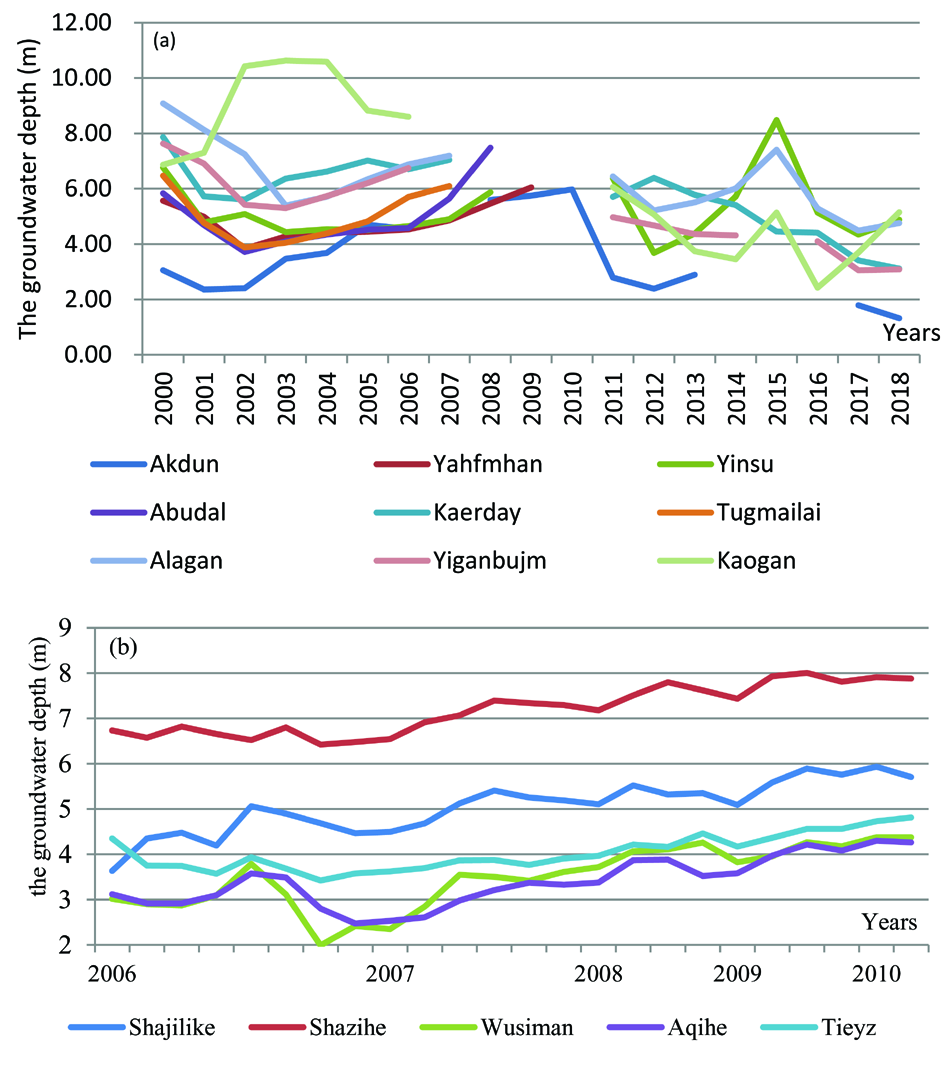

Supplement: Supplementary file 4 — Supplementary Figure S4. [file 41598_2021_96742_MOESM4_ESM.tif]
